# Editorial: Bionics limb prostheses: Advances in clinical and prosthetic care

**DOI:** 10.3389/fresc.2022.950481

**Published:** 2022-08-18

**Authors:** Laurent Frossard, Silvia Conforto, Oskar C. Aszmann

**Affiliations:** ^1^ Griffith Centre of Biomedical and Rehabilitation Engineering, Menzies Health Institute Queensland, Griffith University, Gold Coast, QLD, Australia; ^2^ BioLab³ - DIIEM - Roma Tre University, Roma, Italy; ^3^ Bionic Laboratory of Extremity Reconstruction and Department of Plastic & Reconstructive Surgery, Medical University of Vienna, Vienna, Austria

**Editorial on the Research Topic** Bionic limb prostheses: advances in clinical and prosthetic care by Frossard L, Conforto S and Aszmann OC. (2022). Front. Rehabilit. Sci. 3:950481. doi: 10.3389/fresc.2022.950481

## Context

### Importance of residuum health

The use of a prosthesis is essential to maintain function and wellbeing of individuals suffering from limb absence (1, 2). Consequently, providers of prosthetic care recommend bespoke interventions to sustain lenient interactions between individuals' residual limb and their prosthesis (3–7). The clinical management of this interface is critical because it greatly affects the residuum health (8).

Residuum health is influenced by intrinsic determinants inherent to personal demographics (e.g., gender, age, weight, and height) and surgical amputation (e.g., length of bone, muscle reassignments, muscle strength, and adipose tissue distribution) and extrinsic determinant-associated attachment (e.g., socket design and direct skeletal attachment) and prosthetic components (e.g., choice and alignment of components, control of the prosthetic joint movements, use of walking aids, and level of activity) (8). In all cases, interactions between intrinsic and extrinsic determinants are critical as residual tissues have limited physiological capacity to withstand direct loading applied by typical socket-suspended prostheses during daily activities (e.g., chafing and rubbing) (5, 9–12). In addition to general neurological residuum and phantom pain, individuals can experience a range of incapacitating neuromusculoskeletal dysfunctions compromising residuum health, such as acute and chronic skin issues, edema, neuroma, tendinitis, muscle contractures, stress fractures, osteopenia, and heterotopic bone growth, which altogether increases the risks of sound lower joints osteoarthrosis, and hyperlordosis (6, 13, 14).

Consequently, satisfactory residuum–prosthesis interface is difficult to achieve and sustain (15). Individuals with compromised residuum health are more at risk to experience unsuccessful prosthetic fitting arrangements (4, 16). Those with healthy residuum are more likely to maximize comfort, stability, and mobility when using a suitable prosthesis. Individuals tend to go up and down between low (e.g., bedridden, use of wheelchair, and two crutches without prosthesis), unsatisfactory (e.g., two crutches with prosthesis, one stick, and independent ambulation with pain), and satisfactory (independent ambulation without pain and participation in recreational and professional activities) levels of activity depending on their satisfaction with prosthetic fitting, functional abilities, and need for aids (17–20). Individuals are often trapped going back and forth between unsatisfactory and satisfactory health states depending on pain level with the prosthesis (19, 21). Pain leads to frequent, and too often permanent, prosthesis abandonment (22–24). Altogether, repeated episodes of care addressing prosthetic fitting generate great personal distress and heavy socioeconomic burdens (e.g., healthcare expenses and work absenteeism) (25–30).

### Emergence of new bionic solutions

In the last few decades, we have witnessed promising developments in the production of bionic limb solutions that could possibly alleviate, separately or altogether, some of the residuum health and fitting issues (31–34). Some innovations provide better prosthetic attachment through osseointegrated implants that could either extend the residuum limb and facilitate socket fit (e.g., endoskeletal implant) or protrude the skin to allow the fitting of bone-anchored prostheses (e.g., endoskeletal-exoskeletal implant) (19, 35, 36). Other innovations aim predominantly to reduce pain and improve control of the prosthetic limbs, including regenerative peripheral nerve interfaces, targeted muscle reinnervation (TMR), agonist–antagonist myoneural interface, and sensory feedback (31, 37–40).

Altogether, these emerging bionic bone-anchored prostheses could dramatically alleviate socket-related issues and improve intuitive usage of artificial limbs (33, 41–43). Early evidence of the clinical outcomes of these new interventions has indicated that they have, altogether, the potential to engender life-changing benefits (e.g., body image, sitting comfort, osseoperception, pain reduction, prosthetic control, walking ability, and health-related quality of life) (44–47).

### Need for more information about rehabilitation and prosthetic care bionic solutions

Reports of scientific advances of a particular solution tend to focus primarily on the design of interface between the body and the hardware (e.g., osseointegrated implants and electrodes), screening process (e.g., eligibility criteria), surgical techniques (e.g., number of stages and reinnervation matrices), fitting and design of prosthetic components (e.g., microprocessor-controlled joints and control algorithms) as well as short- to long-term outcomes (e.g., physical tasks and health-related quality of life) (48–52).

Although critical to successful clinical outcomes, rehabilitation procedures (e.g., training exercises) and prosthetic fitting recommendations (e.g., setting of components) for new solutions are often areas of continuous development and, therefore, are under-reported (53–59). The level of understanding and acceptance of pre- and postoperative clinical care may vary between interventions for lower or upper bionic limbs.

More information is needed to elucidate the relationships between surgical procedure, clinical care, prosthetic fitting, and outcomes of current and emerging interventions (e.g., efficacy and safety) that are critical for establishing an evidence-based reasonable, and eventually best, standard of care for current and future bionic solutions.

## Contribution

### Scope of the research topic

Initially, we identified a need for more information about:

• Preoperative interventions that could possibly maximize surgical and medical outcomes of bionic limb solutions (e.g., screening process, strength, and reconditioning, stretching program).
• Postoperative intervention following surgical insertion of osseointegrated implants (e.g., rehabilitation programs, prescription of loading progression, monitoring of loading exercises, design of static and dynamics load-bearing exercises, strength, and conditioning).
• Postoperative intervention after targeted muscle reinnervation (e.g., extraction of physiological signal, development of classifiers, design of fine and/or gross motor control training exercises, training for intuitive control).
• Fitting of bionic and/or bone-anchored prostheses (e.g., choice and alignment of prosthetic components, training with microprocessor-controlled joint units, fall prevention program).
• Short- and long-term outcome measures of efficacy and safety of bionic and/or bone-anchored prostheses extracted from standardized and non-standardized instruments (e.g., physical tasks and self-reported surveys).
• Quantitative evaluation of functional recovery with techniques based on kinematics and dynamics and on the processing of myoelectric signal of the non-amputee limb to study adaptation and recovery strategies also aimed at the optimal choice of prosthesis.

It will be unrealistic to expect that this Research Topic alone will outline the current “state-of-the-art” on these topics. Therefore, we decided to gather a series of highly focused articles presenting forthcoming ideas and concepts as well as preliminary data about current and emerging bionic solutions.

### Outline of key contributions

This Research Topic features a total of 10 articles written by 54 authors (39% females and 61% males) from 23 institutions across 7 countries. It presents one Perspective, Review, Case Report, Brief Research Report, and six Original Research articles.

As detailed in Table 1, six manuscripts involved individuals with transtibial, transfemoral, hip disarticulation, and transhumeral amputations. Two other basic studies used cadavers and animal specimens. Six manuscripts focused on socket interface and three locked at the design of a percutaneous part, the osseointegration process, and the surgical procedure for direct skeletal attachment specific to bone-anchored prostheses. Four studies sought to improve safety of prosthetic care, more particularly reduction of fall, improvement of osseointegration, and reduction in the infection of future osseointegrated implants. Eight studies aimed at improving efficacy, particularly mobility and function, reduction of phantom and residuum pain, and control of prosthesis.

**Table 1:** Key descriptors of the studies presented in this Research Topic.

	**Number of studies**		Raschke (2022)	Taylor et al. (2022)	Bohart et al. (2022)	Borkowska et al. (2022)	Bresslerf et al. (2022)	Kannenberg et al. (2022)	De Marchis et al. (2022)	Finucane et al. (2022)	Bachini et al. (2022)	Boesendorfer et al. (2022)
	**(#)**	**(%)**										
Population	** **											
Cadavers	**1**	**10**	** **	×								
Animal	**1**	**10**	** **		×							
Human	**7**	**70**	** **			×	×	×	×	×	×	×
Able-bodied	**1**	**10**	** **			×						
Amputees	**6**	**60**					×	×	×	×	×	×
Amputation	** **											
All level	**1**	**10**	** **				×					
Lower Limbs	**7**	**70**	** **									
Transtibial	**3**	**30**	** **	×				×	×			
Transfemoral	**3**	**30**	** **						×	×	×	
Hip disarticulation	**1**	**10**	** **							×		
Upper limb	**2**	**20**	** **									
Transhumeral	**2**	**20**		×								×
Attachment	** **											
Socket	**6**	**60**	** **				×	×	×	×	×	×
Bone-anchored prosthesis	**2**	**20**		×	×							
Contribution	** **											
Safety	**4**	**40**	** **	×	×					×		
Fall	**1**	**10**	** **							×		
Osseointegration	**2**	**20**	** **	×	×							
Infection	**1**	**10**	** **		×							
Efficacy	**8**	**80**	×			×	×	×	×	×	×	×
Mobility and function	**5**	**50**	** **					×	×	×	×	×
Phantom pain	**2**	**20**	** **				×				×	
Residuum pain	**1**	**10**	** **					×				
Control	**1**	**10**				×						

The bold values represent the number of articles focusing on the key descriptors in the rows expressed in number of articles and percentage of whole articles (100% = 10 manuscripts published).

### Overview of new bionic solutions

Raschke (2022) wrote an introductory review that provided critical insights into the historical developments of the prosthetic technology and practices within the greater context of successive industrial revolutions (Industry 1.0 to Industry 4.0). Raschke shared her astute perspective on the expected benefits of the current industry revolution. The unfolding Industry 4.0 is characterized by the convergence of physical, digital, and biological systems that support the creation of smart technology and cyber-physical systems enabling innovative bionic bone-anchored prostheses (e.g., advanced manufacturing, additive manufacturing, data analytics, augmented reality, simulation, horizontal/vertical integration, cybersecurity, cloud computing, and the industrial internet). Raschke also highlighted the importance of health economic assessments to determine the balance between the costs and the benefits of these innovations (25).

Taylor et al. (2022) used cadaveric mechanical testing, medical imaging, and finite-element analyses of humeri and tibia to improve the design of the percutaneous osseointegration docking system for direct skeletal prosthetic limb attachment. The translation of the exact system from the humerus to the tibia may not be suitable because of differences in impaction force and stress distribution. Each type of implant must be designed following a specific shape and mechanical constraints.

Bohart et al. (2022) used a porcine model to develop an infection-free integration between the skin and a percutaneous part of skin and bone integrated pylon for direct skeletal attachment of lower limb prostheses. Injections of botulinum toxin into the four thigh muscles of the distal thigh of the left hind leg were sufficient to provide noticeable immobilization the skin’s movement around the implant by the fourth week after the procedure. Injections of botulinum toxin might limit skin movements around a percutaneous part of an implant, thereby possibly reducing postoperative risks of infection.

Borkowska et al. (2022) performed a randomized cross-over study within able-bodied participants to assess the capacity of a new haptic sleeve to improve mechanotactile feedback. This study locked at changes in weak, normal, and strong grasp using visual, haptic, or combined feedback. The mechanotactile feedback provided by the haptic sleeve effectively improve grasping tasks and reduced energy expenditure.

Bresslerf et al. (2022) asked clinicians and end users to complete a System Usability Scale survey and semistructured interview to validate a new computer-assisted limb assessment (CALA) tool that can standardize documentation and visualization of phantom limb sensations and pain and quantify the patient’s body image. CALA allowed for an accurate description and quantitative documentation of phantom limb pain. This capacity to analyze, monitor, and report sensation and pain information can help to close the gap between the therapist's conception and the patient's perception of phantom limb sensation and pain.

Kannenberg et al. (2022) analyzed the outcomes of an online survey completed by 46 individuals with transtibial amputation to determine whether anecdotal reports on reduced musculoskeletal pain and improved patient-reported mobility were isolated occurrences or reflect a common experience in powered prosthetic ankle–foot users. Users reported improvements in mobility and reduction of sound knee and amputated side knee pain when using powered prosthetic ankle–foot compared with passive feet. However, a substantial proportion of powered prosthetic ankle–foot users also reverted to passive feet.

De Marchis et al. (2022) performed a multimodal prosthetic gait assessment using a series of kinematic, kinetic, and electrophysiological datasets collected on individuals with different types of amputations and prosthetic components for a project funded by the Italian Worker’s Compensation Authority. This study showed the importance of analyzing movement neural control and mechanical actuation of prosthetic limb as a whole rather than through segregated analyses focusing specific aspects. Multimodal prosthetic gait assessment can facilitate a more effective design of prostheses and therapies for patients fitted with conventional and new bionic limbs.

Finucane et al. (2022) asked individuals with a unilateral transfemoral or knee disarticulation amputation to follow new training (i.e., verbal, visual, tactile cueing, and patient education) to improve functional mobility (i.e., level-ground walking, stair climbing, incline walking, and sit-to-stand transitions) with a powered knee and ankle prostheses. This study provided new training techniques that can help individuals fitted with lower limb prostheses to take advantage of these powered devices and achieve their desired clinical outcomes.

Bachini et al. (2022) asked an individual with transfemoral amputation to wear four prosthetic interfaces stimulating specific areas of the residual limb (e.g., rigid and a semirigid socket with and without a focal pressure) to investigate if socket design can influence phantom sensations. Phantom sensations were different during distinct phases of the walking gait cycle depending on the four interfaces and led to changes in some gait spatiotemporal parameters. Phantom sensations were modulated by the prosthetic interface and could provide natural somatosensory information dynamically varying with gait phases.

Boesendorfer et al. (2022) reported the experience and outcomes of an individual who opted for an elective arm amputation to solve the lack of function due to obstetric brachial plexus injury. The participant showed a distinct improvement of function and high wearing times of the prosthesis at follow-up assessment. Selected patients who experience severe neurological deficit of biologic hand function might benefit from the elective amputation and subsequent restoration with the bionic hand.

## Next steps

### Sparking discussions

As highlighted by Raschke (2022), the successful development of bionic solutions integrating physical, digital, and biological systems will occur through a multitude of small increments. This Research Topic contributes to this global effort as it identifies knowledge gaps while, hopefully, sparking discussions about these new concepts capable of advancing clinical and prosthetic care of bionic limb prostheses.

### From concept to standard of care

These articles should motivate more teams to engage in formalized research and publications further advancing these innovations. Accumulation of evidence through registered clinical trials will be required to facilitate clinical adoption and subsequent acceptance as standard of prosthetic care. Robust evidence will be required to overcome what Harris (2016) described as the “decline effect” (e.g., Initial strong results of new treatments tend to fade overtime with subsequent independent and stronger studies) (60). This will be critical to convince public and private healthcare funding bodies to support a particular innovation, particularly with the emergence of the fee-for-device business model (e.g., hospital, work cover, and insurance) (25–30).

### Toward a global ecosystem

These clinical and prosthetic care innovations will contribute to the formation of a global ecosystem where a set of organizations and services will integrate the value chain of these bionic solutions through various commercial models. This emerging ecosystem will include providers of prosthetic solutions and administrators of healthcare organizations. More importantly, consumers will be at the heart of the ecosystem through involvement in the co-design of innovations and influence of consumers’ advocates. Involving all stakeholders will critical to warrant that these bionic innovations, indeed, improve safely the life of growing population of individuals suffering from limb loss worldwide.
